# Testing an Attachment-Based Parenting Intervention-VIPP-FC/A in Adoptive Families with Post-institutionalized Children: Do Maternal Sensitivity and Genetic Markers Count?

**DOI:** 10.3389/fpsyg.2018.00156

**Published:** 2018-02-19

**Authors:** Lavinia Barone, Virginia Barone, Antonio Dellagiulia, Francesca Lionetti

**Affiliations:** ^1^Department of Brain and Behavioral Sciences, University of Pavia, Pavia, Italy; ^2^Molecular and Developmental Medicine Department, University of Siena, Siena, Italy; ^3^Department of Psychology, Università Pontificia Salesiana Rome, Italy; ^4^Department of Biological and Experimental Psychology, Queen Mary University of London, London, United Kingdom

**Keywords:** VIPP-FC/A parenting intervention, RCT, adoption, emotional availability, neurobiological markers

## Abstract

This study investigated the effectiveness of a newly integrated version of an intervention targeting adoptive mothers’ positive parenting for promoting children’s emotional availability, by testing the moderating role of both two maternal genetic polymorphisms (i.e., 5HTTLPR and DRD4-VNTR) and emotional availability-EA on intervention outcomes. Mothers with their children (*N* = 80; *M*_age_ = 42.73 years, *SD* = 3.79; *M*_age_ = 33.18 months, *SD* = 16.83 months) participated in a RCT testing the Video-Feedback Intervention to Promote Positive Parenting and Sensitive Discipline-VIPP-FC/A effectiveness. Mixed effects regression models showed a significant improvement in mother–child EA for the VIPP-intervention vs. the dummy intervention condition, with a moderating role of maternal EA on children’s outcomes. No significant moderating effect was found for the two genetic polymorphisms inquired. Children’s and mother’s outcomes obtained are discussed.

## Introduction

Attachment theory provides one of the most comprehensive frameworks for understanding social and emotional development. Bowlby (1969, 1973) stated that the quality of parent–child relationship, mainly determined by maternal sensitivity, is grounded in the biological basis of becoming attached to primary caregivers, with both biological and environmental determinants contributing to a healthy adaptation. Parents’ ability to be sensitive, responsive, supportive, and emotionally available when interacting with their children, referred to as positive parenting, is to be considered the core feature of any attachment-based intervention programs, with a likely important impact on children’s social-emotional well-being (Juffer et al., 2007; Steele and Steele, 2018).

The objective of positive parenting interventions is to enhance parental strengths, particularly maternal sensitivity and emotional availability, as a means to improve parent–child emotional bond and well-being. The Video-Feedback Intervention to Promote Positive Parenting-VIPP (Juffer et al., 2007) is one of the best known and most extensively validated evidence-based home-visit programs and it has been tested in various populations of at-risk parents and vulnerable children, mostly up to 3–4 years old (Juffer et al., 2017a,b). It is based on attachment theory and consists of a short (up to seven home-visits) and narrowly-focused program designed to improve the parent–child relationship by enhancing parental sensitivity and positive parent–child interactions. This program could be more properly described as a group of interventions as it exists in several versions with different focuses: mother’s sensitivity (VIPP), mother’s attachment representations (VIPP-R) and mother’s sensitivity and sensitive discipline (VIPP-SD). It was originally developed to be used in families with newborn children in their first year of life, and has been then extended to older children and tested in several randomized controlled trials with different target populations (Juffer et al., 2007; Dozier and Rutter, 2016). More recent adapted versions are under investigation: the version for fathers (Iles et al., 2017), for day-care centers (Werner et al., 2016), for twin families (Euser et al., 2016) and for families with late-adopted children up to 6 years old (Barone et al., 2017a).

Parenting interventions are all based on the acknowledgment that parenting is a challenging undertaking, and we do know that adoptive parents are faced with the highly demanding task of developing and consolidating an attachment bond with a child whose earlier rearing context has been often neglectful or abusive (Muhamedrahimov and Grigorenko, 2015). In this regard, a recent meta-analysis of Lionetti et al. (2015) has shown that only a minority of children reared in institutions is able to develop a secure attachment with professional caregivers, with most of the children (82%) being classified as disorganized or insecure. Insecure attachment patterns particularly characterize children who spent more than 1 year in institution, thus arriving in the new family beyond infancy (van den Dries et al., 2009). Besides this data, research has also reported a significant improvement in attachment quality following adoption placements (van IJzendoorn and Juffer, 2006), with a substantial contribution played by adoptive parents’ emotional availability in fostering children’s emotional and social adjustment (Barone et al., 2017b).

According to these findings, a main issue pertains to what kind of parental features allow children reared in adoptive families more likely able to develop healthier socio-emotional abilities. A first factor, largely considered in VIPP interventions, is maternal sensitivity. A construct which is close to sensitivity, named emotional availability-EA, is the one that more properly covers the essential features of VIPP-SD, as it implies the simultaneous consideration of several dyadic dimensions ranging from parental sensitivity to structuring, this latter being a disciplinary dimension to proactively structure the environment in order to allow a smoothly emotional exchange between the mother and the child (see for a recent contribution Saunders et al., 2015).

In order to understand what contributes to positive parenting improvement and to the related children’s well-being, maternal EA thus constitutes an expected factor to be studied. Another relevant factor for testing intervention effectiveness is to analyze the role played by specific neurobiological features. To date, putative candidate genes most often studied as moderators of parenting effects on children’s development are those that regulate dopamine, serotonin, and oxytocin systems (DRD4, DRD2, COMT, SLC6A4, OXTR). Among these, two polymorphisms have been studied more extensively than others as candidate “susceptible genes,” i.e., as genes associated with an increased sensitivity to the impact of the environment (see meta-analytic studies of Bakermans-Kranenburg and van Ijzendoorn, 2011); the 43 bp insertion/deletion – 5HTTLPR – in the promoter region of the serotonin transporter gene SLC6A4 (e.g., Cents et al., 2014; Baptista et al., 2017), and the variable number of tandem repeats-VNTR – in the dopamine receptor gene DRD4 (Bakermans-Kranenburg and van Ijzendoorn, 2007). Studies focusing on the role of DRD4-VNTR and 5HTTLPR have shown that carriers of 7 repeat-allele of the DRD4-VNTR gene and carriers of the short allele variant of 5HTTLPR genetic marker were more susceptible to the environment quality, including a change in environment due to an intervention on positive parenting (e.g., Bakermans-Kranenburg and van Ijzendoorn, 2011; Kochanska et al., 2011; Belsky et al., 2015).

Although data on VIPP effectiveness are promising (Juffer et al., 2017a), they also call for further studies able to reach the most vulnerable children by improving their principal caregivers’ emotional availability. To turn to the strengths and main aims of the current study, we tested for the first time with an experimental design the effectiveness of an attachment-based intervention on parental sensitivity and sensitive discipline (VIPP-Foster-Care/Adoption, i.e., VIPP-FC/A, an extended VIPP-SD version for children in adoption and/or in foster care aged up to 6 years) in a potentially challenging context such that of the first year after adoption placement. Furthermore, given the small or modest effect found in the majority of the interventions aimed at enhancing parental sensitivity or in reducing attachment insecurity (Berlin et al., 2016), we added the analysis of two moderating variables – maternal emotional availability on children’s outcomes and maternal neurobiological markers on maternal emotional availability – in order to better explain the factors implied in the individual differences obtained in response to the intervention.

The purpose of this paper specifically was twofold:

(1) To test the effectiveness of the newly extended version of VIPP-SD, i.e., Foster Care and Adoptive Families version- VIPP-FC/A, in promoting maternal positive parenting and children’s emotional adjustment.
(2) To test the effectiveness of the aforementioned intervention by assessing the possible moderating role of both maternal genetic markers and the additive contribution of maternal emotional availability on outcomes.

We hypothesized that:

(1) VIPP-FC/A intervention would promote positive parenting in adoptive mothers, increasing their emotional availability (EA).
(2) Both maternal candidate genetic polymorphisms DRD4-VNTR and 5HTTLPR would moderate the impact of intervention on outcomes. Specifically, we hypothesized that mothers carrying DRD4–7 repeat allele or the 5HTTLPR short allele and involved in the intervention group would present significantly higher improvements in their emotional availability; conversely, carriers with the same allele variants but included in the control condition would decrease their levels of emotional availability after time.
(3) The intervention would be effective in promoting children’s emotional availability, with a key role played by maternal emotional availability.

## Materials and Methods

### Participants

**FIGURE 1:**
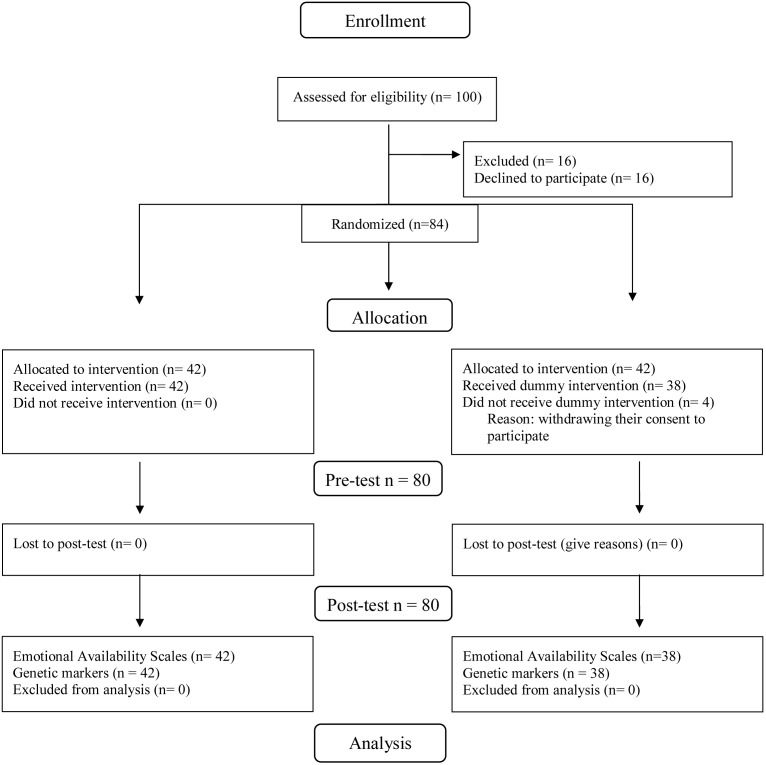
Consort Flow Diagram of the study’s progress, detailing participant numbers during recruitment, inclusion, randomization.

Eighty adoptive mothers (*M*_age_ = 42.73, *SD* = 3.79) with their internationally adopted children, who gave written informed consent to participate in the study (see **Figure 1**), took part to the study.

Mothers had about 16 years of education (*M* = 16.92; *SD* = 2.35), were all Caucasian, predominantly Italian (95%). Children’s mean age at arrival was 33.18 months (*SD* = 16.83 months, age range 1–68 months) and children’s mean age at assessment was 43.15 (*SD* = 15.70, age range 17.45–75 months). 36 of them (46.75%) were female.

### Measures

#### Emotional Availability Scales 4th Edition (EAS; Biringen, 2008)

The scales were used to code dyadic emotional availability between mothers and children at pre and post-intervention. The measure was applied by reliably trained researchers at intake into the study and within 2 weeks from intervention completion, by videotaping 15 min of mother’s and child’s dyadic play. The EAS includes four seven-point scales for parenting behavior: (a) sensitivity, (b) structuring, (c) non-intrusiveness, and (d) non-hostility and two scales for child’s behavior: (a) responsiveness and (b) involvement. A summary score for both positive parenting and child’s EA is obtained by computing the mean value among the EA scales.

#### DNA Collection, Extraction, and Genotyping

Buccal swabs were collected during the first home-visiting and stored at -20°C until DNA was extracted. DNA was extracted using the kit IQ form Promega following manufacturer’s instructions during the first home-visiting. For genotyping of the VNTR in exon 3 of the DRD4 gene (ref seq. NM_001045), PCR was performed using the primers 5′-AGGACCCTCATGGCCTTG HEX-conjugated and 5′-GCTCATGCTGCTGCTCTACT. PCR was performed as follows: 1 cycle of 5 min at 94°C, followed by 10 cycles of 45 s at 94°C, 30 s at 62°C, 1 min 68°C, 25 cycles of 30 s at 94°C, 30 s 52°C, 1 min at 68°C and a final extension step of 7 min at 68°C. The PCR-amplified DNA fragments were analyzed by loading the PCR product on an ABI PRISM 3130xl Genetic Analyzer.

For amplification of the 5′ regulatory region of Serotonin Transporter gene (Locus Symbol SLC6A4; ref seq. NM_000797), which contains a 43 bp insertion/deletion polymorphism (5HTTLPR), primers 5′-TCCTCCGCTTTGGCGCCTCTTCC 6FAM-conjugated and 5′-TGGGGGTTGCAGGGGAGATCCTG were used. PCR was carried out as follows: 1 cycle of 5 min at 94°C, followed by 10 cycles of 45 s at 94°C, 1 min at 68°C, 1 min at 68°C, 25 cycles of 45 s at 94°C, 45 s 55°C, 1 min at 68°C and a final extension step of 7 min at 68°C. The presence of the Short (S) and Long (L) allele for each sample was determined by loading the PCR product on an ABI PRISM 3130xl Genetic Analyzer. All the PCR were carried out in presence of 0.05% DMSO and 1N betaine starting from 2 μl of genomic DNA.

The DRD4–7 repeat allele was present in 36% of mothers; the 5HTTLPR *s/s* genotype in 19% of mothers.

### Procedure

All mothers with adopted children (*N* = 100), in their family for no more than 1 year before the first research contact, were recruited through national health authority adoption services of several Italian towns. Mothers with their children who agreed to participate (*N* = 84) were randomly assigned (using a random numbers generator to assure that each participant had an equal chance of being placed in any of the two groups) either to a group that would attend the VIPP-SD-FC/A or to a control group which received a dummy intervention.

The intervention group resulted with more subjects than the control group (42 vs. 38) because four mothers who were assigned to the control condition withdrew their consent to participate before the completion of the study. No significant children’s gender difference [X^2^(1) = 1.07, *p* = 0.30] between the intervention and the control group was identified; similarly, there was no statistically significant difference concerning age at assessment between the two conditions, *t*(74.60) = -0.89, *p* = 0.37). All children and mothers (both VIPP-SD-FC/A participants and controls) were visited at home before the intervention taking place and screened for emotional availability using the Emotional Availability scales 4th Edition (Biringen, 2008) over the course of a 15-min parent–child interaction (10-min with toys, 5-min of free play). A maternal saliva sample from which to extract the genetic material was collected during the same home-visit. All mothers with their children then got either the VIPP-SD-FC/A or the dummy intervention. The intervention and control conditions were set up in line with the standard procedure for testing VIPP intervention efficacy reported in the literature (e.g., see Negrão et al., 2014), as described below.

#### Control – Dummy Intervention Condition

Parallel to the intervention sessions, mothers in the control group received six telephone calls from the interveners as a dummy intervention. During these phone calls, interveners discussed general child development themes with mothers, providing them with a similar amount of attention as the mothers in the experimental condition.

No information on positive parenting, attachment or sensitivity was provided. Mothers who requested explicit advice or detailed information were referred to their general practitioner and/or their health service agency.

#### Experimental – VIPP Intervention Condition

Mothers and children in the intervention condition received the VIPP-FC/A intervention. This is a short-term, home-based intervention aimed at enhancing primary caregiver sensitivity and positive attitudes and discipline strategies by using video-feedback. The reviewed protocol is suitable for children aged between 18 and 72 months old and involves seven home-visits: an initial session to collect a baseline video of parent–child interaction and six intervention sessions where the intervener gives feedback using the previous week video visit, as well as input on positive parenting techniques. Specifically, the FC/A revised version is an extension of the already tested VIPP-SD and put special attention to children’s even light responsive cues, to modes of affect sharing, to physical contact attempts, to children’s seeking help actions and to possible still present indiscriminate friendliness behaviors; that is all key behaviors that are considered eligible intervention targets for adopted children social-emotional adjustment.

#### Interveners and Assessment Coders

All interveners were trained and certified for adherence in accordance with the VIPP training guidelines. Furthermore, all interveners were trained in the new revised version of VIPP-FC/A. The same interveners who provided the intervention conducted the home-visiting and phone calls with adoptive mothers in the control condition. To avoid contamination of data, different coders – all reliably trained on EA scales and blind to group membership and to mothers’ genotyping – rated the pre-test and post-test measures.

### Plan of Analysis

#### Effect of the VIPP Intervention on Mothers’ Outcomes, i.e., Positive Parenting, and Testing of the Moderating Role of Candidate Genetic Markers

The efficacy of the intervention was tested using mixed-effects regression models with random intercepts. Specifically, for testing intervention efficacy in promoting maternal positive parenting, the following models were performed and compared by using the Bayesian Information Criterion (BIC) and the Akaike Information Criterion (AIC): the Model 0, i.e., the null model (with intercept only); Model 1, i.e., the model where the time (1 = pre-intervention; 2 = post-intervention) is assumed as predictor of changes; Model 2, i.e., the time variable plus intervention condition as predictor of changes in mothers’ positive parenting; Model 3, i.e., the intervention model (with the interaction between time elapsed and the experimental condition explaining changes in mothers’ positive parenting). Finally, for testing the moderating role of the genetic polymorphisms under inquiry, we added the gene susceptible condition as a moderator in the analysis. In Model 4a the 5HTTLPR S/S condition was tested, in Model 4b for the DRD4–7 repeated condition, and Model 4c was used to test the condition of at least one susceptible gene (i.e., either DRD4 or 5HTTLPR susceptible markers).

### Effect of the VIPP Intervention on Children’s Outcomes, i.e., Emotional Availability-EA, and Testing of the Contribution of Mother’s EA

In order to explore the role of the VIPP intervention on children’s outcomes across time, the following models were compared: the Model 0, i.e., the null model (with intercept only); Model 1, i.e., the model where the time elapsed (1 = pre-intervention; 2 = post-intervention) is assumed as predictor of changes; Model 2, i.e., the time variable plus intervention condition as predictor of changes; Model 3, i.e., the intervention model, with the interaction between time elapsed and the experimental condition explaining changes. Finally, for testing the contribution of mothers, we added mother’s emotional availability in the regression model as predictor (Model 4), and we repeated the same models adding the contribution of children’s gender (Model 5) and age at adoption placement (Model 6). Graphical representations were used for further exploring moderating and additive effects. The model with the lowest AIC and BIC was assumed as the best model fitting our data, and its parameters were thus explored. Analyses were conducted with the statistical software R; the lme4 package was used for mixed-effects model and the lmerTest for computing the *p*-values of main and interaction effects of the best model selected.

## Results

### Descriptive Analyses and Measures Psychometric Properties

At the pre-intervention assessment, correlation between maternal EA scales ranged from *r* = 0.40 for the association between sensitivity and non-hostility scales to *r* = 0.88 for the association between sensitivity and structuring scales. Also, a one-factor model fitted the data well [X^2^(1) = 3.589, *p* = 0.166, CFI = 0.994, TLI = 0.983, RMSEA = 0.02], thus allowing the mean of the four scales to be used as a summary Emotional Availability-EA score for positive parenting (see also Negrão et al., 2014). Similarly, the correlation between the two children’s EA scales (responsiveness and involvement) was 0.93, thus supporting the use of a EAS summary score for children in regression model. Inter-coder reliability for randomly chosen observations (10% of all data) was good (*r* = 0.81).

At the baseline assessment, no statistically significant difference was reported pertaining to the mean level of positive parenting of mothers belonging to the control condition group (*M* = 5.39, *SD* = 0.86), *vs*. the experimental condition group [*M* = 5.46, *SD* = 0.88; *t*(76.69) = -0.379, *p* = 0.705], whereas children belonging to the control group tended to score higher than children belonging to the VIPP intervention condition [i.e., *M* = 4.93 vs. 4.36, *t*(73.98) = 2.07, *p* = 0.04]. Emotional Availability in children did not correlate with gender (*r* = 0.01, *p* = 0.96) neither with age at adoption placement (*r* = 0.11, *p* = 0.35).

Maternal distribution of the genotypes for the candidate polymorphisms in the VIPP intervention and in the control group was as follows: in the control condition, 16 subjects (42%) presented the 48 bp VNTR marker for the DRD4 dopaminergic system (i.e., any 7+), and 8 (21%) the 42 bp ins∖del marker (i.e., S/S) for the 5HTTLPR serotoninergic system. In the intervention condition, 13 subjects (31%) presented the 48 bp VNTR marker, and 7 (21%) the 42 bp ins∖del marker. No significant difference was identified between the intervention and the control group nor for DRD-VNTR marker distribution [X^2^(1) = 0.130, *p* = 0.72] neither fir the 5HTTLPR 42 bp ins∖del marker distribution [X^2^(1) = 0.671, *p* = 0.20]. Also, the difference between positive parenting at the pre-intervention assessment in mothers carrying the 48 bp VNTR marker for the DRD4 dopaminergic system and in mothers without the putative susceptible genes was not significant [*t*(57.87) = -0.88, *p* = 0.38]. The same applied for the 42 bp ins∖del marker for the 5HTTLPR serotoninergic system [*t*(21.03) = 0.97, *p* = 0.34].

### The Role of the VIPP Intervention and the Failed Contribution of the Two Genetic Variables in Predicting Maternal Positive Parenting

The BIC and AIC identified the model representing the interaction between time and intervention condition as the best of the four in predicting an increase in maternal Positive Parenting (**Table 1**), whereas none of the models including the genetic variables improved the model fit. Coefficients of the best model selected and associated *p*-values are reported in **Table 2**. At post-intervention assessment (Time 2), the dummy intervention group had a positive parenting score similar to that at the pre-intervention assessment (Time 1) (at baseline, *M* = 5.39, *SD* = 0.86, at post-intervention assessment *M* = 5.38, *SD* = 0.98), whereas the VIPP intervention group showed a significant increase in Positive Parenting (at baseline, *M* = 5.46, *SD* = 0.88; EAS at post-intervention assessment *M* = 6.05, *SD* = 0.61). The two groups, which did not differ at the baseline, were significantly different at the post-intervention assessment time point [*t*(69.74) = -3.72, *p* < 0.001]. The differential score between post and pre intervention assessment was -0.01 (*SD* = 0.85) for the dummy intervention group and 0.59 (*SD* = 0.66) for the VIPP intervention group, respectively. The association between the intervention condition and the differential score was *r* = 0.39, suggesting a moderate effect size for this VIPP intervention version effectiveness.

**Table 1:** VIPP-FC/A intervention effects on maternal Emotional Availability.

Model	BIC	AIC
Null model – random intercept	406.16	396.93
Model 1 – time^a^	405.37	393.07
Model 2 – time + condition^b^	407.38	392.00
Model 3 – time × condition	**402.75**	**384.31**
Model 4a – Time × condition × 5HTTLPR	425.51	394.76
Model 4b – Time × condition × DRD4–7 repeat	422.41	391.66
Model 4c – Time × condition × at least one susceptible gene	424.60	393.84


*Models comparison of mixed effects regression models. For Model 4a, the distinction between 5HTTLPR S/S vs. S/L and L/L was considered; for Model 4b, the distinction between at least one DRD4–7 repeat allele vs. others was considered; for Model 4c the distinction between at least one DRD4–7 repeat or 5HTTLPR SS condition vs. others was considered. ^*a*^1 = pre-intervention condition; 2 = post-intervention. ^*b*^1 = control condition; 2 = intervention condition.*

**Table 2:** Coefficients of the best model selected (i.e., Model 3).

	*B*	*SE*	*t*(df)	*p-*Value
Intercept	5.40	0.14	41.27 (115.90)	<0.001
Time^a^	-0.01	0.12	-0.05 (78.00)	0.960
Condition^b^	0.07	0.17	0.39 (115.90)	0.697
Time × condition	0.60	0.17	3.48 (78.00)	<0.001


*^*a*^1 = pre-intervention; 2 = post-intervention.^*b*^1 = control condition; 2 = intervention condition.*

**FIGURE 2:**
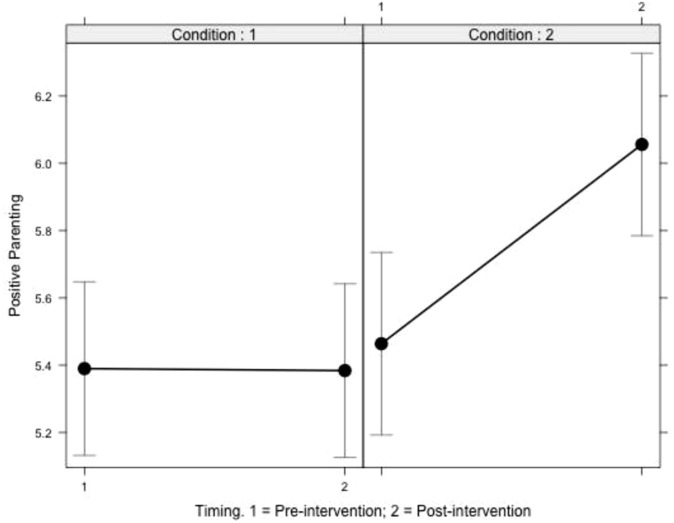
Intervention condition effect on positive parenting. Condition 1 refers to the dummy intervention; Condition 2 to the VIPP intervention condition.

**Figure 2** reports interaction effects and specifically maternal Positive Parenting scores at Time 1 (pre-intervention assessment) and at Time 2 (post-intervention assessment), showing that for the control condition, maternal Positive Parenting did not improve over time, or even worsened slightly, whereas for the intervention condition an improvement was observed.

### The Role of the VIPP Intervention and the Contribution of Maternal Positive Parenting in Predicting Children’s EA

The BIC and AIC identified the models representing the interaction between time and intervention condition, and including the contribution of maternal Positive Parenting as predictor, as the best model in predicting an increase in children’s EA (**Table 3**). Coefficients of the best model selected and associated *p*-values are reported in **Table 4**. At post-intervention assessment (Time 2-T2), children belonging to the control condition group had a score comparable to that reported at the pre-intervention assessment (EA at T1, *M* = 4.93, *SD* = 1.15, EA at T2, *M* = 5.03, *SD* = 1.33), whereas children belonging to the intervention condition group showed a significant increase (EA at T1, *M* = 4.36, *SD* = 1.35; EA at T2, *M* = 5.49, *SD* = 1.08). The differential score of children’s EA between post and pre intervention assessment was 0.09 (*SD* = 1.12) for the control group, and 1.13 (*SD* = 0.84) for the VIPP intervention group, respectively. The association between the intervention condition and the differential score was *r* = 0.46, suggesting a moderate effect size for intervention effectiveness. The degree of change in EA was negatively associated with children’s age at adoption placement (*r* = -21) suggesting that those arrived later were more slightly resistant to change, but the difference was not significant (*p* = 0.07) and the association was low in effect size. Also, no significant association between the differential score of children’s EA and gender was identified (*r* = -0.05).

**Table 3:** VIPP-FC/A intervention effects on children.

Model	BIC	AIC
Null model – random intercept	524.55	515.33
Model 1 – time^a^	512.34	500.03
Model 2 – time + condition^b^	518.31	502.92
Model 3 – time × condition	505.36	486.91
**Model 4 – Time × condition + maternal EA**	**403.79**	**382.26**
Model 5 – Time × Condition + Gender	482.62	461.62
Model 6 – Time × Condition + Age at adoption placement	501.73	480.38


*Models comparison of mixed effects regression models. ^*a*^1 = pre-intervention; 2 = post-intervention. ^*b*^1 = control condition; 2 = intervention condition.*

**Table 4:** Coefficients of the best model selected (i.e., Model 3).

	*B*	*SE*	*t*(df)	*p-*Value
Intercept	-0.418	0.44	-0.941 (153.87)	0.348
Time^a^	0.10	0.12	0.86 (76.60)	0.393
Condition^b^	-0.647	0.188	-3.435 (113.68)	<0.001
Time × condition	0.443	0.08	2.55 (82.26)	0.013
EAS mother	0.443	0.17	12.62 (153.84)	<0.001


*^*a*^1 = pre-intervention; 2 = post-intervention. ^*b*^1 = control condition; 2 = intervention condition.*

**FIGURE 3:**
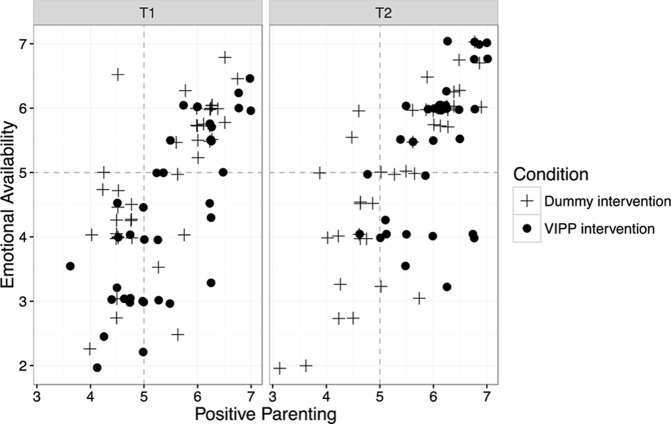
Associations between maternal positive parenting scores and children’s emotional availability scores in the VIPP and dummy intervention condition at the pre-intervention assessment (T1, box on the left) and at the post-intervention assessment (T2, box on the right). Each point in the figure represents a mother–child dyad.

To sum up and graphically represent the results obtained we can observe that at the post-intervention assessment (T2), children who attended the VIPP intervention with their mothers showed a clear increase in their emotional availability scores and, furthermore, mother–child dyads belonging to the intervention condition tend to present higher scores of both positive parenting and emotional availability if compared to mother–child dyads belonging to the dummy intervention condition(**Figures 2, 3**).

## Discussion

The present study explored the effectiveness of a recently integrated version of an attachment-based intervention for promoting positive parenting – i.e., the Foster Care and Adoptive Families adaptation- VIPP-FC/A, suitable for children up to 6 years of age. Its effectiveness was investigated by analyzing first the main effect of the intervention condition and second the possible moderating effect of two maternal genetic polymorphisms – i.e., DRD4 and SLC6A4 – on intervention outcomes (i.e., maternal positive parenting) within a longitudinal experimental design. We then tested the specific contribution of mothers’ positive parenting on children’s outcomes (i.e., emotional availability). As expected, results showed that VIPP-FC/A worked well with this family typology and significantly improved the ability of both children and mothers who took part in the intervention to behave in a sensitive and emotionally available manner during their home interactions, whereas no significant improvement and indeed a slight worsening was observed for children and mothers who did not attend it. Our results showed that the best model fitting the data was that implying the intervention vs. the control condition, with a medium effect size for both mothers and children’s outcomes (*r* = 0.39 and *r* = 0.46, respectively), values that are in line with meta-analytic findings indicating a satisfactory effect size for the group of VIPP interventions (Juffer et al., 2017b). This is of particular interest given the datum that, in general, parenting interventions show a peculiar constraint in failing to reach an acceptable and univocally interpreted effect size in the trials implemented, also mainly showing an only modest effect (Berlin et al., 2016). To our knowledge the present study is the first to apply the new integrated version of the VIPP intervention in a population of adoptive parents of late-adopted children, up to 6 years old. Given the increasing number of late adoptions, and the importance of preschool years for children’s social-emotional education, testing the efficacy of this version of the intervention will play a role in closing the gap between parenting programs dealing with infants’ and those dealing with older children’s developmental issues.

With regards to the possible moderating effect of specific molecular genetics on parenting, several features of the current study are particularly notable in the context of prior research on this topic. First we used an experimental design for testing our hypothesis, being aware of the more compelling evidence of the importance of the interplay between genes and environment provided by these studies if compared to correlational ones; intervention trials provide a unique opportunity to explore gene–environment interplay, particularly in the context of differential susceptibility hypothesis (Ellis et al., 2011; van Ijzendoorn and Bakermans-Kranenburg, 2015). The randomization of participants to trial groups allows for careful evaluation of the role of genetics as well as the role of environment. This appears particularly relevant because of the manner in which the environment is controlled, thus limiting potential gene–environment correlation, one of the main pitfall of this kind of research (Latendresse et al., 2017). Second, in order to manipulate the environmental variable we compared two conditions (i.e., control vs. intervention) by conducting an evidence-based intervention with a possibly challenging population such as it is that of adoption mothers in their first year after adoption placement. Contrary to our expectation, no significant moderating role of genotyping on intervention outcomes was detected, thus rejecting our second hypothesis claiming their possible specific moderating role.

The absence of an effect observed in our study for either dopamine or serotonin neurotransmitters adds further evidence to the claim of the sometimes unsuccessful outcomes obtained targeting “candidate genes” underlying specific behaviors (Farrell et al., 2015) and is likely due to two main factors. First, the sample size of our study. Data analyses could hardly detect genetic moderating effects when small frequencies in some cell sizes for certain genotypes are present (see for a recent discussion Jolicoeur-Martinau et al., 2018). Second, when a clear main effect is detected and ranges are from moderate to strong, as it was for the intervention condition of our sample, an interaction term including other variables may lead to non-significant findings because of the presence of an already main effect. Nevertheless, our micro-trial could supplement important data to the field by adding outcomes including maternal genetics, up to now actually lacking. Our findings didn’t confirm previous results of those studies that found a moderating effect of the two genes polymorphisms considered on attachment or related constructs outcomes (e.g., Bakermans-Kranenburg and van Ijzendoorn, 2011; Cents et al., 2014; Baptista et al., 2017) and thus add further evidence on the mixed nature of data in this research field so far. As recently highlighted (see, e.g., Luijk et al., 2011; Leerkes et al., 2017), there is still little consistent evidence for the role of candidate genes in predicting the outcomes of attachment or close constructs as sensitivity. Also, research in G × E studies are recently moving toward the simultaneous analysis of multiple genes, suggesting that a more extended analysis of multiple genetic markers of sensitivity may need to be performed if we want to reach a more in depth understanding of individual differences in response to environmental stimuli (Keers et al., 2016).

Another important even if expected finding was that, beyond the lack of a genetic bond, maternal emotional availability played a significant role on children’s outcomes and, relevant, this was not the case for other variables as children’s gender or age at adoption placement. According to the data obtained, the VIPP intervention affected children’s emotional availability toward their mothers, increasing their ability to be more responsive and more able to actively involve the parent in the interaction. Worthy of note is that this was true for both girls and boys and that only a small difference was detected concerning the age at adoption placement, with older children showing less change after the intervention completion.

Next to strengths, our study also has some limitations, which constitute future research directions. First, although our multisite recruitment efforts and the added value of the experimental design implemented, our sample size was likely limited for detecting possible effects of the two moderating genes under investigation. Future studies should replicate our findings and contribute to future meta-analyses able to collect similar small sample studies. Second, human parenting is more complex than accounted for by influences on maternal sensitive discipline; far less explored are the genetic mechanisms underlying the competence of caregivers other than mother, including fathers. Future studies could also include fathers, possibly replicating the outcomes obtained. Despite these limitations our research offers a reliable contribution to a very innovative and challenging field of research by presenting an experimental microtrial design targeting very definite mechanisms involved in parenting and children’s well-being, also providing critical information on how a specific I × E interaction may or may not affect adoptive mothers and children in the context of an attachment-based intervention aimed at preventing later children’s behavioral problems.

## Ethics Statement

All procedures performed involving human participants of the study were approved by the Ethical Committee of the Department of Brain and Behavioral Science of Pavia University and of IUSS-Scuola Universitaria Studi Superiori Pavia, Italy and were in accordance with APA ethical standards. Written informed consent was obtained from all families (both parents) participating into the study. All subjects gave written informed consent in accordance with the Declaration of Helsinki.

## Author Contributions

LB and FL developed the study design. LB supervised data collection, contributed in the analyses planning, and wrote the paper. FL and AD performed data analysis and contributed to the final draft. VB supervised the analysis of genetic data.

## Conflict of Interest Statement

The authors declare that the research was conducted in the absence of any commercial or financial relationships that could be construed as a potential conflict of interest.
